# Equatorial Non-muscle Myosin II and Plastin Cooperate to Align and Compact F-actin Bundles in the Cytokinetic Ring

**DOI:** 10.3389/fcell.2020.573393

**Published:** 2020-09-25

**Authors:** Joana Leite, Fung-Yi Chan, Daniel S. Osório, Joana Saramago, Ana F. Sobral, Ana M. Silva, Reto Gassmann, Ana X. Carvalho

**Affiliations:** ^1^Cytoskeletal Dynamics Lab, Instituto de Investigação e Inovação em Saúde (i3S), Universidade do Porto, Porto, Portugal; ^2^Cytoskeletal Dynamics Lab, Instituto de Biologia Molecular e Celular (IBMC), Universidade do Porto, Porto, Portugal

**Keywords:** actomyosin contractility, cytokinesis, non-muscle myosin II, contractile ring, *C. elegans*, cortical flows, plastin/fimbrin

## Abstract

Cytokinesis is the last step of cell division that physically partitions the mother cell into two daughter cells. Cytokinesis requires the assembly and constriction of a contractile ring, a circumferential array of filamentous actin (F-actin), non-muscle myosin II motors (myosin), and actin-binding proteins that forms at the cell equator. Cytokinesis is accompanied by long-range cortical flows from regions of relaxation toward regions of compression. In the *C. elegans* one-cell embryo, it has been suggested that anterior-directed cortical flows are the main driver of contractile ring assembly. Here, we use embryos co-expressing motor-dead and wild-type myosin to show that cortical flows can be severely reduced without major effects on contractile ring assembly and timely completion of cytokinesis. Fluorescence recovery after photobleaching in the ingressing furrow reveals that myosin recruitment kinetics are also unaffected by the absence of cortical flows. We find that myosin cooperates with the F-actin crosslinker plastin to align and compact F-actin bundles at the cell equator, and that this cross-talk is essential for cytokinesis. Our results thus argue against the idea that cortical flows are a major determinant of contractile ring assembly. Instead, we propose that contractile ring assembly requires localized concerted action of motor-competent myosin and plastin at the cell equator.

## Introduction

Animal cytokinesis relies on the assembly and constriction of an actomyosin contractile ring at the cell equator, which pinches the plasma membrane inwards, ultimately separating the two daughter cells (reviewed in Leite et al., 2019). The contractile ring is a circumferential array of filamentous actin (F-actin), non-muscle myosin II (hereafter myosin) and actin-binding proteins, including formins and F-actin crosslinkers, such as α-actinin, fimbrin/plastin (hereafter plastin), anillin and septins (reviewed in Green et al., 2012). At cytokinesis onset, RhoA activation at the cell equator triggers the recruitment and activation of downstream effectors to assemble the contractile ring, particularly diaphanous-related formins (Prokopenko et al., 2000; Großhans et al., 2005; Watanabe et al., 2010; Melak et al., 2017) and Rho kinase, which promote the assembly of linear F-actin and the activation of myosin, respectively (Matsumura, 2005; Vicente-Manzanares, 2009). Experimental evidence from electron microscopy in marine invertebrate and newt eggs and cultured mammalian cells suggests that the contractile ring is a thin layer of closely packed F-actin of mixed polarities (Schroeder, 1968, 1970, 1972; Arnold, 1969; Selman and Perry, 1970; Sanger and Sanger, 1980; Maupin and Pollard, 1986; Mabuchi et al., 1988; Henson et al., 2017) surrounded by a mostly branched network (Henson et al., 2017). How F-actin and myosin accumulate and organize into a circumferential array at the cell equator to form the contractile ring is a central question that remains to be fully resolved. Two main mechanisms have been implicated: equatorial compression due to cortical flows and local RhoA-dependent *de novo* assembly at the equator. According to the former, both F-actin and myosin are advected by flow from the poles towards the equator due to a tension differential between these regions. The persistent convergence at the equator creates a region where filaments are constantly compressed and aligned (White and Borisy, 1983; Salbreux et al., 2009; Turlier et al., 2014; Khaliullin et al., 2018). According to the latter, F-actin and myosin are directly recruited to the equatorial cortex from the cytoplasm, leading to local alignment of F-actin bundles (Kimura et al., 1996; Bement et al., 2005; Vavylonis et al., 2008; Uehara et al., 2010; Spira et al., 2017). The relative contribution of these two mechanisms to cytokinesis has been difficult to ascertain, because flows are myosin-dependent (Reymann et al., 2016) yet myosin perturbations may prevent cytokinesis simply because myosin-dependent force generation at the equator is essential for equatorial deformation and continued furrow ingression (Davies et al., 2014; Osorio et al., 2019). While actomyosin cortical flows towards the cell equator during cytokinesis have been described in several experimental systems, including *C. elegans* (Reymann et al., 2016; Khaliullin et al., 2018; Singh et al., 2019), they are less evident in some mammalian cells and are absent in yeast (Cao and Wang, 1990; Murthy and Wadsworth, 2005; Wu and Pollard, 2005; Vavylonis et al., 2008; Laplante et al., 2015). It remains to be determined whether cortical flows actually drive assembly of a contractile ring or whether they are simply a consequence of the tension differential that arises from equatorial stimulation and polar relaxation.

The involvement of F-actin crosslinkers (hereafter crosslinkers) in contractile ring assembly remains poorly explored. Depending on their size and structure, crosslinkers can form tight and parallel F-actin bundles or loosely crosslinked F-actin networks with distinct architectures and mechanical properties (Faix et al., 1996; Mavrakis et al., 2014; Srivastava and Robinson, 2015). A common feature is that crosslinkers provide interconnectivity, which is important for contractility of actomyosin networks (Ennomani et al., 2016; Descovich et al., 2017). To date, the only crosslinker whose perturbation is known to negatively impact cytokinesis in *C. elegans* embryos is the plastin PLST-1. A *plst-1* loss-of-function mutant decreased F-actin network connectivity and resulted in short-range and erratic F-actin cortical flows (Ding et al., 2017). Plastin is a globular protein that can assemble F-actin into tightly packed bundles with uniform or mixed polarity (Bretscher, 1981; Matsudaira et al., 1983; Skau et al., 2011). Plastin localizes to dense F-actin bundles and is particularly abundant in the cytokinetic ring (Ding et al., 2017), but whether it contributes to the organization and/or stabilization of F-actin bundles in this region is unknown.

We previously generated *C. elegans* expressing motor-dead mutants of myosin (NMY-2) and showed that myosin motor activity is essential for cytokinesis in the one-cell embryo (Osorio et al., 2019). In this study, we use these *nmy-2* mutants, as well as a *plst-1* mutant, to explore the role of cortical flows during cytokinesis in the one-cell *C. elegans* embryo. Our data from quantitative live imaging assays for cortical F-actin and myosin flows, contractile ring assembly and furrow ingression, myosin turnover dynamics, and F-actin bundle alignment at the cell equator, collectively support the idea that contractile ring assembly and furrow ingression requires the concerted action of motor-competent myosin and plastin at the cell equator and is largely independent of compressive cortical flows.

## Results

### Long-Range Cortical F-actin Flows Are Dispensable for Contractile Ring Formation and Furrow Ingression

**FIGURE 1:**
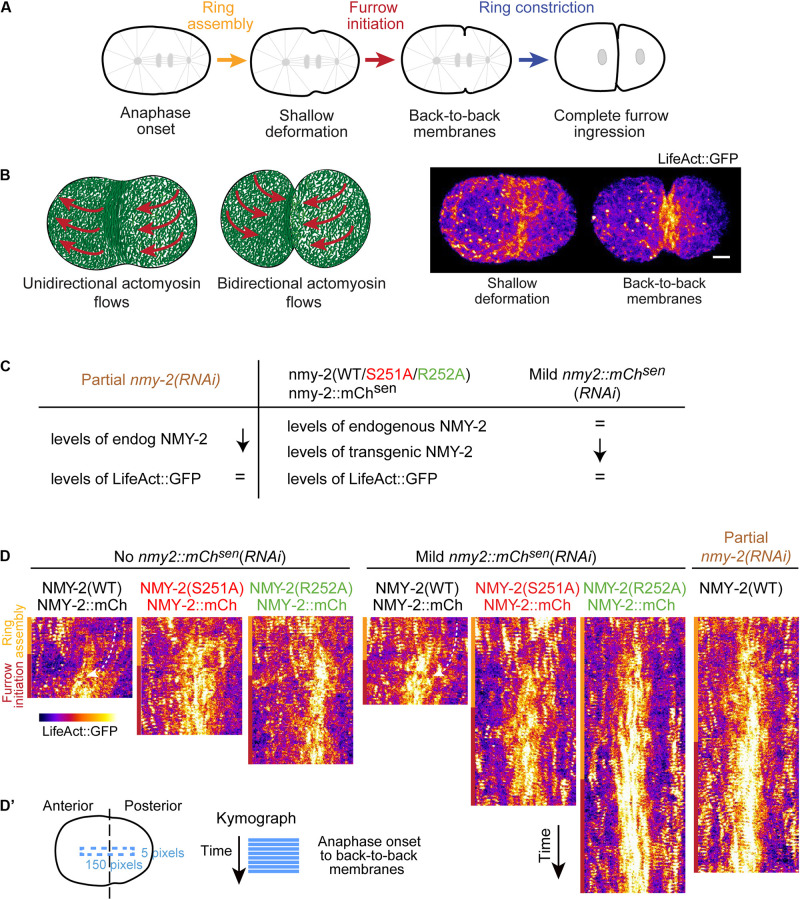
Perturbation of myosin motor activity or reduction of myosin levels lead to significantly reduced cortical F-actin flows. **(A)** Schematic of cytokinesis. Reference points used for measurements of ring assembly and furrow initiation intervals are indicated. Ring assembly: time interval between anaphase onset and the formation of a shallow equatorial deformation, when contractile ring components are being recruited to the cell equator. Furrow initiation: time interval between shallow deformation and the folding of the plasma membrane into a back-to-back configuration. Ring constriction: time interval between back-to-back membrane configuration and full furrow ingression. **(B)** Stills of cortical images of an embryo expressing LifeAct::GFP at shallow deformation and furrow initiation with a schematic illustrating the transition from unidirectional cortical flows during ring assembly to bidirectional flows starting at the onset of ring constriction. **(C)** Perturbations used and their effect on the levels of endogenous and transgenic NMY-2 and LifeAct::GFP relative to controls with no RNAi, as described in Osorio et al. (2019). **(D)** Kymographs of LifeAct::GFP cortical flows along the longitudinal axis in one-cell embryos for the conditions indicated in **(C)**. Yellow and red bars correspond to ring assembly and furrow initiation time intervals, respectively. Curved arrows indicate posterior-anterior flow direction. **(D’)** Schematic illustrating the region of the embryo used for the kymographs in **(D)**. Scale bar, 5 μm.

To investigate the importance of compressive cortical flows for assembly and constriction of the contractile ring (Figures 1A,B), we analyzed F-actin cortical flow profiles in one-cell embryos co-expressing endogenous motor-dead NMY-2(S251A) or NMY-2(R252A) and a transgenic wild-type version of NMY-2 that is mCherry-tagged and RNAi-sensitive (NMY-2::mCherry^*sen*^; Figure 1C) (Osorio et al., 2019). NMY-2(S251A) and NMY-2(R252A) are unable to translocate F-actin but retain the ability to bind F-actin *in vitro* (Osorio et al., 2019), and embryos expressing endogenous motor-dead myosins fail cytokinesis after penetrant RNAi-mediated depletion of NMY-2::mCherry^*sen*^ (Osorio et al., 2019). Mild RNAi-mediated depletion of NMY-2::mCherry^*sen*^ slows but does not prevent cytokinesis.

F-actin flow analysis was performed using transgenic LifeAct::GFP, whose cortical levels are unaffected by myosin inhibition (Osorio et al., 2019). We chose LifeAct::GFP (Silva et al., 2016; Chan et al., 2019; Osorio et al., 2019) rather than LifeAct::mKate2 (Reymann et al., 2016), because in our hands one-cell embryos expressing LifeAct::mKate2 exhibited delayed furrow initiation and had a significantly reduced contractile ring constriction rate when compared to embryos expressing other fluorescent contractile ring components (Supplementary Figure S1A). In contrast, cytokinesis kinetics were normal in embryos expressing LifeAct::GFP. Moreover, the cortical distribution of LifeAct::GFP, but not that of LifeAct::mKate2, was similar to the cortical distribution of endogenous PLST-1::GFP (Supplementary Figure S1B), which binds all F-actin pools during cytokinesis (Ding et al., 2017).

**FIGURE 2:**
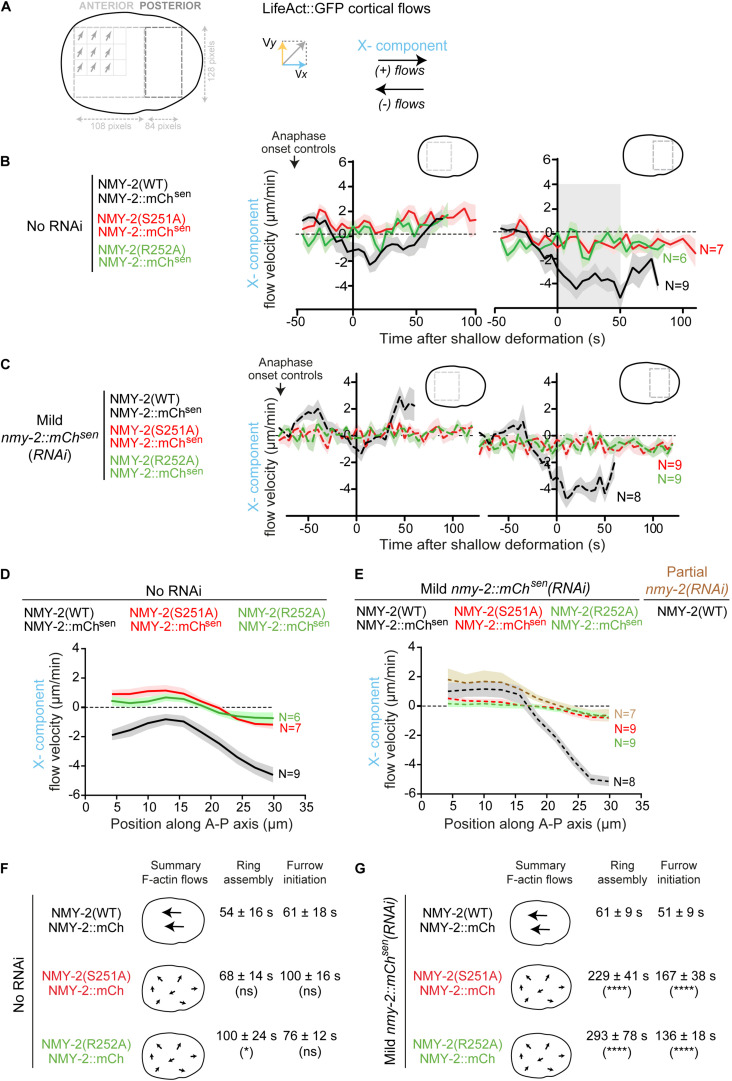
Contractile rings assemble and cytokinesis completes in a timely manner when long-range cortical actin flows are drastically reduced. **(A)** Schematic illustrating how cortical LifeAct::GFP flows were analyzed by particle image velocimetry (PIV; see more details in “Materials and Methods” section). **(B,C)** Longitudinal (X-component) flow velocity (mean ± SEM) over time in the anterior or posterior half of embryos. Curves were aligned to the time of shallow deformation. N is the number of embryos analyzed. Period of consistently strong, posterior-anterior directed flows is indicated in gray. **(D,E)** Longitudinal (X-component) velocity profiles of Lifeact::GFP cortical flows along anterior-posterior (A-P) axis averaged for a period of 50 seconds starting at shallow deformation. Flow profiles show mean X-component velocity ± SEM. N is the number of embryos analyzed. **(F,G)** The schematic on the left summarizes overall flow intensity and directionality (X-component). Ring assembly and furrow initiation time intervals (mean ± SD) are indicated. Statistical significance in (D), (E) was determined using two-way ANOVA for repeated measures, and in (F), (G) using one-way ANOVA followed by Bonferroni’s multiple comparison test; *P < 0.05, ****P < 0.0001, ns, not significant.

Kymographs of the cortical region between the onset of anaphase and the establishment of a back-to-back plasma membrane configuration revealed F-actin flows directed from the posterior towards the anterior pole in control embryos (Figures 1A–D). Even without depletion of transgenic NMY-2::mCherry, flows were barely detectable in *nmy-2(S251A)* and *nmy-2(R252A)* embryos, nor were they evident in embryos partially depleted of endogenous wild-type myosin (Figures 1C,D). To quantitatively analyze cortical flows, we performed particle image velocimetry (PIV) analysis (Figure 2A). Flow velocity was calculated by averaging the X- or Y-components (anterior-posterior and dorsal-ventral, respectively) of the velocity vectors in two regions positioned over the anterior and posterior half of the embryo, for each time point between anaphase onset and back-to-back membrane configuration (Figures 2B,C). Like reported in Singh et al. (2019), the Y-component was essentially absent when embryos were imaged without compression in an open chamber (Supplementary Figures S2A,B; Singh et al., 2019), yet these embryos completed cytokinesis with kinetics that were indistinguishable from those in embryos imaged under compression (Supplementary Figure S2C). We therefore focused our attention on the X-component. Negative velocity values indicate posterior-anterior oriented flows and positive velocity values indicate anterior-posterior oriented flows (Figure 2A). In agreement with a prior study (Reymann et al., 2016), control embryos presented anterior-directed F-actin cortical flows that started just prior to equatorial shallow deformation, peaked shortly thereafter, and remained high for approximately 50 seconds (Figure 2B). In contrast to controls, most vectors detected in *nmy-2(S251A)* and *nmy-2(R252A)* embryos were extremely weak, had no coherent directionality, and did not increase in magnitude after equatorial shallow deformation (Figures 2B,F). This flow profile was observed regardless of whether transgenic NMY-2::mCherry^*sen*^ was mildly depleted by RNAi or not (Figures 2C,G). We further analyzed the flow velocity profiles by averaging the X-component of the vectors for each position along the length of the embryo during the first 50 seconds after equatorial shallow deformation, when flows in control embryos showed coherent direction and velocity (region highlighted in gray in Figure 2B). In control embryos, flow velocity was higher in the posterior half of the embryo, reaching a maximum velocity of −4.5 μm/min and decreasing toward the anterior pole (Figure 2D). In *nmy-2(S251A)* and *nmy-2(R252A)* embryos the flow profile was flat with or without depletion of transgenic NMY-2::mCherry^*sen*^, reaching a maximum velocity of −1 μm/min (Figures 2D,E). We conclude that endogenous motor-dead myosin has a dominant inhibitory effect on cortical flows in embryos expressing transgenic NMY-2::mCherry^*sen*^. Similarly weak flow profiles were observed in embryos after partial depletion of endogenous wild-type NMY-2 (Figure 2E and Supplementary Figures S3C, S6C).

Analysis of cytokinesis kinetics showed that in the presence of transgenic NMY-2::mCherry^*sen*^, *nmy-2(S251A)* and *nmy-2(R252A)* embryos exhibited only minor delays in contractile ring assembly and furrow initiation despite severely impaired cortical flows (Figure 2F). In contrast, mild depletion of transgenic NMY-2::mCherry^*sen*^ in these embryos resulted in substantial delays in cytokinesis and occasional cytokinesis failure, while cortical flows were similar to those observed in the absence of RNAi treatment (Figure 2G). We conclude that there is no obvious correlation between perturbed cortical flows and delayed cytokinesis. This argues against the idea that persistent, unidirectional actin cortical flows are a major determinant of cytokinesis.

### Cortical Flows Are Dispensable for Accumulation of Myosin in the Ingressing Furrow

To further probe the significance of cortical flows, we sought to evaluate the possibility that flowing myosin generates tension that pushes F-actin to the equator. We first compared cortical flows of LifeAct::GFP and NMY-2::GFP using PIV analysis, which showed that F-actin and myosin flow toward the equator in a concerted manner (Supplementary Figures S3A,B): unidirectional cortical flows from the posterior pole toward the anterior side of the embryo initiate shortly after anaphase, and flow velocities increase linearly until a maximum velocity is reached and maintained for ∼50 seconds. This is followed by a period of bidirectional flows starting at constriction onset, in which actin and myosin flow from both poles towards the ingressing furrow (positive flow velocity in the anterior and negative flow velocity in the posterior side of the embryo).

**FIGURE 3:**
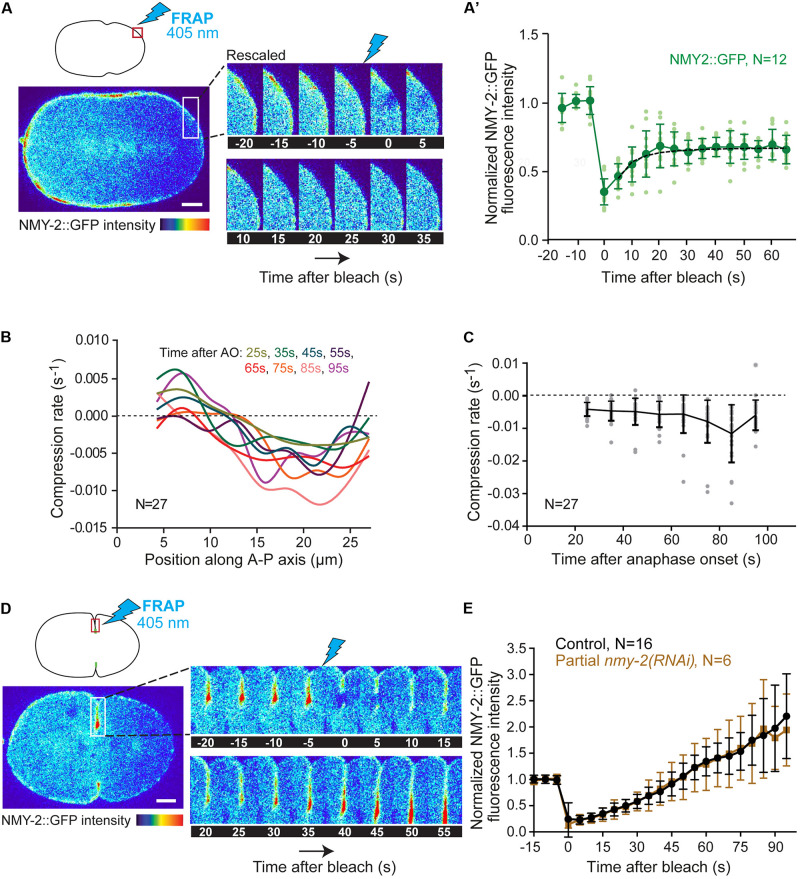
Cortical myosin turnover and flow-induced compression rates. **(A)** Schematic showing the photobleached region at the posterior pole of the embryo at shallow deformation (top left) and selected images from a time-lapse series showing NMY-2::GFP fluorescence over time pre- and post-bleaching. **(A’)** Quantification of NMY-2::GFP fluorescence recovery after photobleaching at the posterior pole. Data from individual embryos (light green) and the mean ± SD (dark green) are plotted. The black dashed line is the single exponential fitting curve from which the half time of recovery and myosin *k*_*off*_ were extrapolated. **(B)** Average compression rate by flow calculated as the spatial derivative of the longitudinal flow profiles for each position along the anterior-posterior (A-P) axis of the embryo from 25 to 95 seconds after anaphase onset (AO). Compression is uniform in the equatorial region (15–25 μm). Colors represent different time intervals as indicated. **(C)** Average compression rate by flow over time after anaphase onset for the region of equatorial compression (15–25 μm). Data from individual embryos (gray) and the mean ± SD (black) are plotted. **(D)** Schematic showing the photobleached region just behind the tip of the ingressing furrow after back-to-back membrane configuration is achieved (top left) and selected images from a time-lapse series showing the NMY-2::GFP fluorescence over time pre- and post-bleaching. **(E)** Quantification of NMY-2::GFP fluorescence recovery after photobleaching in the ingressing furrow in control and NMY-2 depleted embryos. The mean ± SD is plotted. N is the number of embryos analyzed. Scale bars, 5 μm.

Next, we determined myosin turnover at the cell cortex outside the equator. We reasoned that if flowing myosin actively slides F-actin, myosin turnover should be relatively slow, since myosin is known to be stabilized at sites of increased tension such as the contractile ring (Reichl et al., 2008; Srivastava and Robinson, 2015). We photobleached NMY-2::GFP at the posterior polar region at shallow deformation and followed signal recovery during the period of contractile ring formation and compaction. By the time of shallow deformation, some myosin has already been cleared from the posterior pole (Supplementary Figures S4A,B), and therefore confounding effects of flows on the measurement of signal recovery after photobleaching should be reduced. Fluorescence recovery data could be fitted with a single exponential from which we extrapolated a half time of recovery (τ_*half*_) of 4.9 ± 2.4 seconds (Figures 3A,A’). As a control, we photobleached a region of identical size in the posterior cytoplasm, for which signal recovery was significantly faster (τ_*half*_ of 2.6 ± 1.8 seconds; *P* < 0.05; Supplementary Figures S5C,D). Assuming a diffusion-dominated recovery profile for cortical myosin, we calculated a dissociation constant (*k*_*off*_) of 0.07 ± 0.03 s^–1^ (equation 1 in “Materials and Methods” section). Fast myosin turnover in the polar cortex indicates that flowing myosin is in constant exchange with the cytoplasmic pool before reaching the cell equator.

We next determined the rate at which cortical flow compresses F-actin towards the equatorial region. We measured the flow X-component as a function of the position along the anterior-posterior axis for 10 seconds intervals, from 25 seconds until 95 seconds after anaphase onset, and estimated the compression rate by flow for each time interval by calculating the spatial derivative of the flow velocity for each position (Figure 3B). Compression was found to be uniform in the equatorial region (15–25 μm). The average compression rate for the equatorial region increased over time, reaching a maximum of 0.012 ± 0.009 s^–1^ 85 seconds after anaphase onset (Figure 3C). As the maximum compression rate is slower than the rate of myosin dissociation from the cortex, these data argue against the idea that flowing myosin is responsible for F-actin bundle compression at the cell equator.

To confirm this, we assessed myosin turnover in the ingressing furrow. We photobleached a small region just behind the tip of the ingressing furrow and followed NMY-2::GFP signal recovery in the moving region as the furrow continued to ingress. NMY-2::GFP fluorescence intensity in the bleached region increased until the end of ingression without reaching a plateau (Figure 3E). Similar results were obtained when the entire division plane was photobleached at shallow deformation (Supplementary Figures S5A,B). These demonstrate that myosin continues to accumulate in the contractile ring during furrow ingression. Myosin could accumulate by incoming flow, by localized recruitment from the cytoplasm, or both. To distinguish between these possibilities, we examined embryos partially depleted of NMY-2, in which the initial unidirectional flows are essentially absent, bidirectional flows are significantly decreased, and cytokinesis still completes (Supplementary Figure S3C). Under these conditions, NMY-2::GFP was still visible at the tip of the furrow, which allowed us to perform the photobleaching experiment. Normalized recovery profiles of NMY-2-depleted embryos were identical to those of non-depleted embryos (Figure 3E). These results support the idea that the contribution of cortical flows to myosin accumulation at the cell equator is minor and that equatorial myosin is therefore primarily recruited from the cytoplasm. Further support for this idea comes from the observation that the amount of myosin that accumulates at the cell equator after anaphase onset is much higher than the amount of myosin that leaves the polar regions (Supplementary Figure S4B).

### The F-actin Crosslinker Plastin Cooperates With Myosin to Align F-actin Bundles at the Division Plane

We previously found that *nmy-2(S251A)* and *nmy-2(R252A)* embryos depleted of transgenic NMY-2::mCherry^*sen*^ accumulated equatorial F-actin bundles with a tilted orientation (Osorio et al., 2019). Based on this observation, we speculated that F-actin crosslinkers could also contribute to the organization of F-actin bundles at the cell equator. The plastin ortholog PLST-1 was a promising candidate, as it localizes in the contractile ring and the surrounding cortex and has been implicated in cytokinesis (Ding et al., 2017).

**FIGURE 4:**
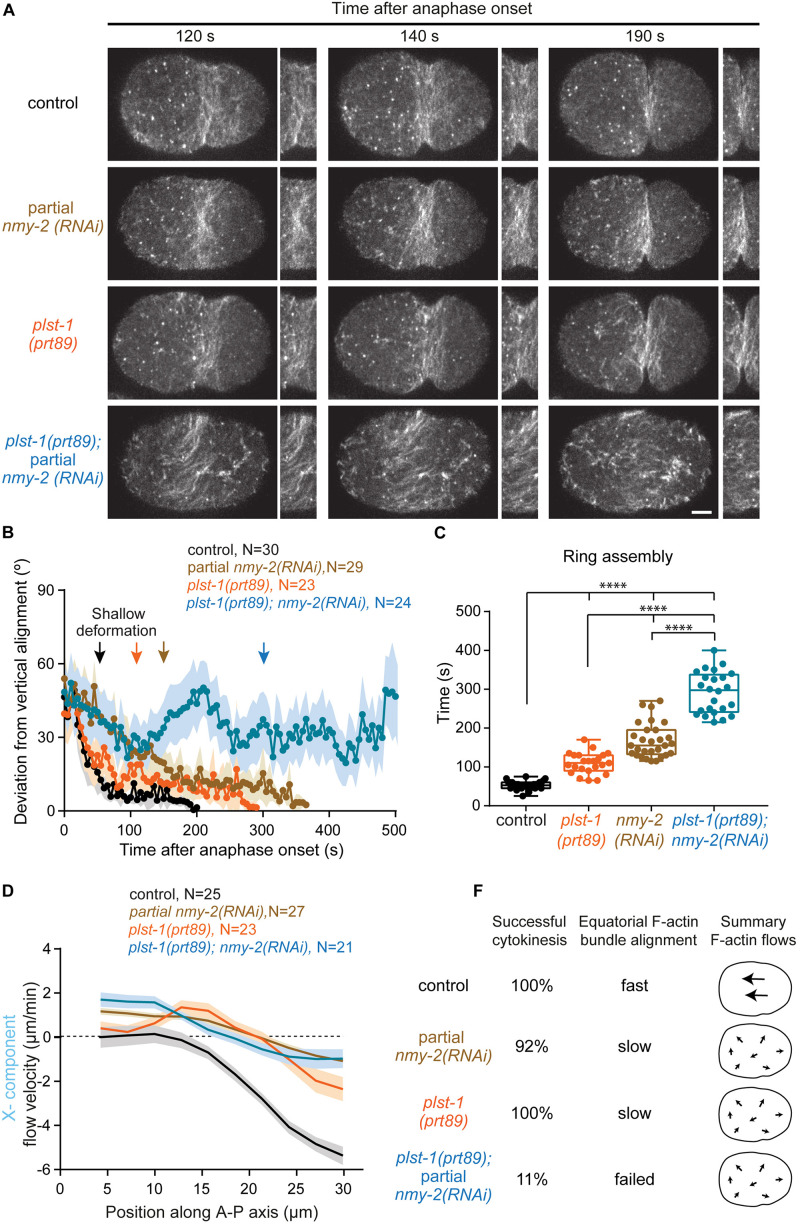
The F-actin crosslinker plastin cooperates with myosin to align F-actin bundles at the division plane. **(A)** Images of the cortical plane of embryos expressing LifeAct::GFP showing the evolution of the equatorial F-actin band relative to anaphase onset. Also see Supplementary Movie S1. **(B)** Deviation from vertical alignment of F-actin bundles at the equatorial cortex relative to anaphase onset (mean ± SEM). **(C)** Boxplots showing the dispersion of contractile ring assembly times. In each box, the central line is the median, the boundaries of the box are the 25th and 75th percentiles; the whiskers extend to include the maximum and minimum values. **(D)** Longitudinal (X-component) velocity profiles of Lifeact::GFP cortical flows along anterior-posterior (A-P) axis averaged for a period of 50 seconds starting at shallow deformation. Flow profiles show mean X-component velocity ± SEM. **(E)** Values on the left indicate percentage of embryos that completed cytokinesis successfully. On the right, a summary of F-actin bundle alignment at the equator and overall flow intensity and directionality (X-component). N is the number of embryos analyzed. Statistical significance of ring assembly times in (C) was determined using one-way ANOVA followed by Bonferroni’s multiple comparison test. ^****^P < 0.0001. Scale bar, 5 μm.

To determine whether NMY-2 and PLST-1 cooperate to compact and align F-actin bundles at the cell equator, we used genome editing to generate a *plst-1* mutant, *plst-1(prt89)*, which contains a premature stop codon in its open reading frame and is homozygous viable, like another previously described *plst-1* mutant (Ding et al., 2017). Using LifeAct::GFP, we quantified F-actin bundle alignment at the equatorial region over time, starting at anaphase onset (Figures 4A,B and Supplementary Movie S1). Analysis of F-actin bundle orientation in control embryos revealed fast bundle alignment perpendicular to the longitudinal axis of the embryo with bundles reaching maximum alignment shortly after shallow deformation (deviation from vertical alignment close to 0°). In embryos partially depleted of NMY-2, F-actin bundle alignment was significantly slower and continued throughout furrow initiation. In *plst-1(prt89)* embryos, F-actin bundle alignment was also slower than in control embryos, with F-actin bundles becoming mostly aligned at shallow deformation and reaching maximum alignment just before furrow initiation. In *nmy-2(RNAi)*;*plst-1(prt89)* embryos, F-actin bundles became completely unstable and failed to align, exhibiting oscillating orientations between 20° and 50° relative to vertical alignment.

In *nmy-2(RNAi)* embryos, median contractile ring assembly was delayed threefold (155 seconds *versus* 53 seconds in controls), but 92% of embryos were able to complete cytokinesis (Figures 4C,E). In *plst-1(prt89)* embryos, contractile ring assembly was delayed twofold (110 seconds), and cytokinesis was successful in all embryos. Strikingly, contractile ring assembly was delayed sixfold (298 seconds) in *nmy-2(RNAi)*; *plst-1(prt89)* embryos, and 89% of embryos failed to initiate furrowing (Figures 4C,E).

We asked whether deficient alignment of F-actin bundles at the cell equator and cytokinesis failure in *nmy-2(RNAi);plst-1(prt89)* embryos could be due to aggravated cortical flows (Figures 4D,E and Supplementary Figure S6). Using PIV analysis of LifeAct::GFP, we determined flow velocities from anaphase onset until the back-to-back membrane configuration (Supplementary Figure S6C). In *plst-1(prt89)* embryos, cortical flows were decreased and erratic compared to controls. There were no periods of unidirectional or bidirectional flows, as the cortex was constantly moving back and forth in opposite directions (Supplementary Figures S6A,C). In *nmy-2(RNAi)*; *plst-1(prt89)* embryos, there was no further aggravation of flow profiles compared to the single perturbations. Analysis of flow velocity along the anterior-posterior axis for the period of persistent unidirectional flow, as defined in control embryos (50 seconds starting at shallow deformation), revealed that *plst-1(prt89)* embryos showed an intermediate profile between that of control and *nmy-2(RNAi)* embryos, with a maximum flow velocity at the posterior pole of −2.5 μm/min (Figure 4D). In *nmy-2(RNAi)*; *plst-1(prt89)* embryos, flow velocities were similar to those observed in *nmy-2(RNAi)* embryos. We further investigated the correlation between vectors as a function of the distance between them for the period of persistent unidirectional flows (Supplementary Figure S6B; equation 2 in “Materials and Methods” section). The closer the cosine similarity is to 1, the higher the correlation between vectors and the more coherent the flow directionality. In control embryos, the cosine similarity was close to 1 for neighboring vectors and decayed gradually as the distance between vectors increased, approaching a minimum of 0.7 for vectors located on opposite poles. In *nmy-2(RNAi)* embryos, the cosine similarity decayed significantly faster, reaching a minimum of approximately 0.2 for the maximum distance between vectors. The correlation was even worse for *plst-1(prt89)* embryos, with minimum values approaching 0, meaning that vectors were pointing in opposite directions. In *nmy-2(RNAi)*; *plst-1(prt89)* embryos, cosine similarity was identical to that in *plst-1(prt89)* embryos, revealing similar problems in flow directionality. We conclude that cytokinesis failure in *nmy-2*(*RNAi);plst-1(prt89)* embryos is unlikely to be caused by an aggravation of cortical flows.

We also inspected the behavior of NMY-2::mCherry signal in the single and double perturbations (Supplementary Movie S1). Interestingly, we observed that residual myosin in *nmy-2(RNAi);plst-1(prt89)* embryos accumulates at the equator more slowly than in *nmy-2(RNAi)* embryos. These results suggest that PLST-1, in addition to its role in promoting connectivity throughout the cell cortex, has a localized role at the cell equator independently of cortical flows. We conclude that localized synergistic action of NMY-2 and PLST-1 at the division plane is critical for F-actin alignment and compaction into a circumferential array that is capable of sustaining furrow ingression.

## Discussion

### Unidirectional Long-Range Cortical Flows Play a Minor Role During Cytokinesis

Several models have been proposed to explain how the cell shape changes that occur during cytokinesis are triggered. The first theories date back to the 1950s and considered cleavage as an integrated process involving reorganization of the entire cell surface. While Mitchison (1952); Mitchison and Swann (1955), and Swann and Mitchison (1958) proposed that cytokinetic furrow formation was a passive event resulting from an active expansion at the poles that would spread toward the equator, Marsland proposed that the furrow region was itself contractile, independently of the poles (Marsland, 1950, 1956a,b). Wolpert’s astral relaxation theory specifically proposed that a negative signal from the astral microtubules would make the poles relax, allowing the equator to contract (Wolpert, 1960). While the molecular mechanisms for polar relaxation have only recently started to be unraveled (Mangal et al., 2018; Chapa-y-lazo et al., 2020), it has become widely accepted that cytokinesis requires a positive signal from the central spindle that activates the small GTPase RhoA, which in turn promotes the accumulation of contractile ring components, F-actin and myosin, at the cell equator. F-actin alignment and accumulation via flow-driven equatorial compression and RhoA-dependent local recruitment are the two main mechanisms thought to drive the assembly of a circumferentially aligned contractile ring.

A recent study showed that F-actin cortical flows are myosin-dependent and that compression by flow, equatorial F-actin alignment, and furrow ingression show similar spatiotemporal dynamics in the one-cell *C. elegans* embryo (Reymann et al., 2016). The authors proposed that persistent, unidirectional compressive flows towards the cell equator dictate the timing of equatorial F-actin alignment and furrow formation. We find that embryos co-expressing similar amounts of endogenous motor-dead and transgenic wild-type myosin have severely decreased F-actin cortical flows, yet contractile ring assembly and furrow initiation still occur in a timely manner, and cytokinesis completes. Lowering the levels of transgenic wild-type myosin in these embryos results in substantial cytokinesis delays with no additional effects on cortical flows. While these results show that both long-range cortical flows and cytokinesis require myosin motor activity, they suggest that cortical flows are neither essential for cytokinesis nor a major determinant of cytokinesis kinetics.

Apart from the cytokinetic furrow, *C. elegans* one-cell embryos also form a transient furrow, which partially ingresses and then regresses, during the period of polarity establishment that precedes the first mitotic division. Unlike cytokinesis, pseudocleavage lacks equatorial accumulation of active RhoA, F-actin and myosin (Mayer et al., 2010; Reymann et al., 2016). Moreover, counter-rotating flows in the two halves of the embryo occur during pseudocleavage initiation that do not occur during cytokinetic furrow initiation (Naganathan et al., 2014). While our results argue that cortical flows play a relatively minor role in furrow formation during cytokinesis, it remains possible that cortical flows are essential for furrow formation during pseudocleavage, as proposed by Reymann et al. (2016).

### Myosin Accumulation at the Cell Equator Occurs Primarily by Recruitment From the Cytoplasm

There is evidence that myosin and other crosslinkers become stabilized and immobilized in regions of increased tension, such as the contractile ring (Reichl et al., 2008; Srivastava and Robinson, 2015). Our experiments of fluorescence recovery after photobleaching and measurements of flow-driven equatorial F-actin compression suggest that the rate of myosin turnover at the cortex outside the cell equator is significantly faster than the rate at which cortical flow compresses the actomyosin network toward the equator. The results agree with findings in *D. discoideum* cells, where cortical myosin did not move all the way to the equator, but rather appeared and disappeared shortly after from the cortex (Yumura et al., 2008). This indicates that flowing myosin may not be able to generate substantial amounts of compressing force. Thus, tension generated by equatorial myosin activity is likely more relevant for the alignment and compaction of F-actin bundles, which is in agreement with sustained RhoA positive signaling at this location. It also indicates that compression and alignment of the equatorial F-actin bundles may occur independently of flows. We show that unidirectional and bidirectional cortical flows can be severely reduced without affecting the recovery profile of photobleached myosin in the ingressing furrow. This suggests that cortical flows may not be the main source of myosin loading onto the contractile ring, in contrast to a previous model (Khaliullin et al., 2018). We propose that myosin accumulates at the furrow primarily by direct recruitment from the cytoplasm to the cell equator and largely independently of cortical flows.

### Plastin-Mediated Bundling and Myosin Motor Activity Are Locally Required for F-actin Alignment at the Division Plane and Deformation of the Equator

**FIGURE 5:**
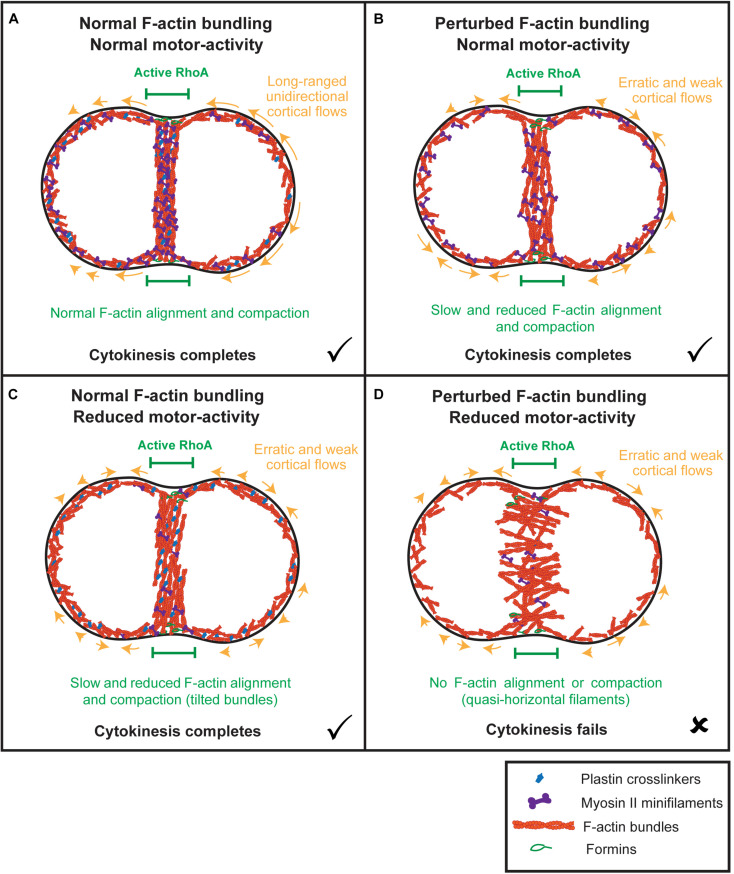
Model: the combined synergistic action of plastin and myosin aligns and compacts F-actin at the division plane independently of actomyosin cortical flows. **(A)** When both plastin and myosin accumulate at the equator, F-actin bundles are properly connected, and myosin is able to efficiently align and compact them into a circumferential array to maximize tension. Early actomyosin flows are unidirectional and long-ranged. **(B)** In the absence of plastin-mediated bundling, myosin struggles to align and compact F-actin bundles, initially spending energy on filament sorting instead of filament sliding. Cortical flows are reduced and erratic and cytokinesis completes with delays in F-actin alignment and contractile ring formation. **(C)** In the presence of plastin-mediated F-actin bundling and decreased myosin levels, F-actin bundles are connected to each other but tilt and struggle to orient along the division plane and to compact because of insufficient motor-activity. Cortical flows are severely reduced and erratic and cytokinesis completes with delays in F-actin alignment and contractile ring formation. **(D)** When both plastin-mediated bundling and myosin levels are decreased, equatorial actin filaments become unstable and start rotating and oscillating, adopting a quasi-horizontal orientation. Cortical flows are reduced and erratic, as in the individual plastin and myosin perturbations. F-actin bundles never align to form the contractile ring and cytokinesis fails.

The contractile ring must produce sufficient force to deform the plasma membrane against the overall cell shape. Actomyosin contractility must therefore be optimized at the cell equator, which involves the organization of F-actin in circumferentially aligned and compacted linear bundles. An F-actin network architecture composed of modules of antiparallel F-actin bundles is optimal for contraction, as filaments are already aligned and oriented such that myosin-dependent filament sliding is facilitated and tension can be readily propagated across the network (Ennomani et al., 2016). It is still unclear, however, whether F-actin bundles in the contractile ring are organized in an antiparallel manner (reviewed in Pinto et al., 2013; Murrell et al., 2015) and whether contractile modules exist (Carvalho et al., 2009; Silva et al., 2016). In fact, *in vitro* reconstituted circular actomyosin networks have been shown to contract in the absence of a modular organization, as long as F-actin crosslinkers are present (Ennomani et al., 2016; Wollrab et al., 2019). Our *in vivo* data show that myosin struggles to align and compact equatorial F-actin at the cell equator without the crosslinking activity of plastin in *plst-1(prt89)* embryos. We envision that plastin facilitates myosin-driven alignment by organizing formin-nucleated linear F-actin into tight bundles, so that tension can be efficiently amplified and propagated throughout the network to rapidly compact it into a circumferential array (Figure 5). Without plastin, myosin may initially spend energy on sorting randomly oriented F-actin instead of productive filament sliding. When myosin levels are reduced in the presence of plastin (partial depletion of NMY-2), F-actin bundles also struggle to align and compact, likely because of insufficient motor activity to produce tension in the network. When both F-actin bundling and myosin levels are reduced in *nmy-2(RNAi);plst-1(prt89)* embryos, equatorial F-actin bundles become unstable and never orient along the division plane. Consequently, contractile ring assembly fails altogether. The enhanced defects in the plastin/myosin double perturbation cannot be ascribed to aggravated cortical flows, since these are already severely affected in the single perturbations, in which a contractile ring assembles and cytokinesis completes. This strongly suggests that myosin and plastin cooperate locally at the cell equator.

Interestingly, the observation that residual myosin in *nmy-2(RNAi)*; *plst-1(prt89)* embryos accumulates at the equator more slowly than in *nmy-2(RNAi)* embryos suggests that plastin-dependent F-actin bundling may stabilize myosin at the equatorial cortex. This is not the first time cooperation between a passive and an active crosslinker has been observed. In *C. elegans*, a previous study demonstrated that plastin provides the connectivity for coalescence and maturation of myosin foci from distant nascent filaments in the cortex, amplifying the range of contractile domains during polarity establishment (Ding et al., 2017). In *D. discoideum*, myosin and the crosslinker cortexillin have been proposed to stabilize each other at sites of increased mechanical load: cortexilin I, as a long-lived crosslinker, anchors F-actin in the network so that myosin can exert more tension, and this promotes accumulation of yet more myosin and cortexillin (Ren et al., 2009). Whether a similar mechanosensing mechanism between myosin and plastin operates during cytokinesis in *C. elegans* embryos is an interesting hypothesis to test in the future.

## Conclusion

In conclusion, while cortical flows may help compress and align F-actin at the equator, our results argue that this is not the main mechanism that drives contractile ring assembly. Instead, we propose that contractile ring assembly requires the localized and concerted action of myosin and the F-actin bundler plastin.

## Materials and Methods

### *C. elegans* Strains

Strains used in this study are listed in Supplementary Table S1 and were maintained at 20°C on nematode growth medium (NGM) plates seeded with OP50 *E. coli*.

NMY-2 motor-dead mutants and the RNAi-sensitive wild-type nmy-2 transgene fused to mCherry are described in a previous study (Osorio et al., 2019). To generate the allele *plst-1(prt89)*, the endogenous locus of *plst-1* was modified using CRISPR/Cas9-mediated genome editing. An extra nucleotide and a *Nhe*I restriction site were introduced to cause a frameshift and premature stop at the beginning of the *plst-1* coding sequence. Note that the *plst-1* sequence in WormBase (version WS276) lacks the first part of the gene corresponding to the EF hands and CH1 domain of the PLST-1 protein (Ding et al., 2017). The premature stop codon that we introduced occurs after amino acid 262 in the CH2 domain of PLST-1 (Ding et al., 2017). The repair template for this modification was the following:

5′*ctctgaaaatccacaaatttccagactgctccgcgacggtgaaactcttgaagatctc* aggcgtctgtccccagaagaaattctcatgAaga**gctagc**tgggttaattatcatttagaacg tgcta*gaactcaacgtcgcctacacaatttcacatcagatattgtcgattcagagatttatac*-3′, where the extra nucleotide is in uppercase, the *Nhe*I restriction site for diagnostic PCR of the genomic edit is in bold, silent mutations introduced to avoid repair template recognition by Cas9 are underlined, and flanking 58 bp homology regions are in italics. Three single guide RNAs (sgRNAs) were chosen using the online tool http://crispr.mit.edu and cloned into the pDD162 vector (Dickinson et al., 2013): 5′-TTCTTCTGGGGACAGACGCCGG-3′, 5′-ATTATCATTTGGAACGTGCTGG-3′ and 5′-AGATGGGTTAATTATCATTTGG-3′. Underlined in the sgRNAs are the PAM sequences, which were not included in the pDD162 vector. The single-stranded repair template (IDT) and pDD162 were mixed together and injected into gonads of young adults N2 hermaphrodites. To facilitate the identification of positive animals, the injection mix also contained a sgRNA and a repair template to generate the R92C mutation in DPY-10, which causes a dominant roller phenotype (Levy et al., 1993; Arribere et al., 2014). To screen for animals carrying the desired modification in the *plst-1* locus, amplification of a plst-1 gene fragment was performed using the primers 5′ CATGGCCTAGAAACCACTTT 3′ and 5′ GCTTCTCGGCTTCATCTAAC 3′, followed by digestion with the *Nhe*I restriction enzyme that recognizes the restriction site present in modified animals only. The modification was confirmed by DNA sequencing. To remove potential off-target mutations, the mutant was subjected to six rounds of outcrossing with N2 animals, before crossing with animals expressing LifeAct::GFP or LifeAct::GFP and NMY-2::mCherry. Although expression of the PLST-1 protein was not assessed by immunoblotting, the defects in *plst-1(prt89)* embryos are similar to those observed for *plst-1(tm4255)*, which is a null mutant (Ding et al., 2017).

### RNA Interference

RNAi-mediated NMY-2 depletion was performed by feeding hermaphrodites with bacteria that express double stranded RNA (dsRNA) targeting *nmy-2*. The dsRNAs nmy-2_RNA#1 and nmy-2_RNA#2 (Supplementary Table S2) are described in Osorio et al. (2019). Expression of dsRNAs in transformed HT115 *E. coli* was induced with 1 mM IPTG and the bacteria were fed to hermaphrodites as described below.

In experiments where specific depletion of transgenic NMY-2::mCherry^*sen*^ was desired [labeled as *nmy-2::mCherry^*sen*^(RNAi)* in Figures 1D, 2C,E,G], nmy-2_RNA#3 was used in GCP22, GCP618 or GCP592 L4 hermaphrodites. Mild depletions (22–26 h of RNAi treatment at 20°C) were used in Figures 1D, 2C,E,F. In experiments whose purpose was to deplete endogenous NMY-2 to an extent that did not preclude cytokinesis [labeled as partial *nmy-2*(*RNAi*)], nmy-2_RNA#1 (29–31 h of RNAi treatment at 20°C) was used in GCP113 L4 hermaphrodites for Figures 3D,E and Supplementary Figure S3C, and nmy-2_RNA#2 (23–27 h of RNAi treatment at 20°C) was used in GCP21, GCP22, and GCP927 L4 hermaphrodites for Figures 1D, 2E, 4A–E, and Supplementary Figure S6.

### Live Imaging

Gravid hermaphrodites were dissected and one-cell embryos were mounted in a drop of M9 (86 mM NaCl, 42 mM Na_2_HPO_4_, 22 mM KH_2_PO_4_, and 1 mM MgSO_4_) on 2% agarose pads overlaid with a coverslip. Live imaging of cytokinesis was performed at 20°C. Images were acquired on a spinning disk confocal system (Andor Revolution XD Confocal System; Andor Technology) with a confocal scanner unit (CSU-X1; Yokogawa Electric Corporation) mounted on an inverted microscope (Ti-E, Nikon) equipped with a 60x NA 1.4 oil-immersion Plan-Apochromat objective, and solid-state lasers of 405 nm (60 mW), 488 nm (50 mW), and 561 nm (50 mW). For image acquisition, an electron multiplication back-thinned charge-coupled device camera (iXon; Andor Technology) was used. For photobleaching experiments, a FRAPPA photobleaching module (Andor Technology) placed between the spinning disk head and the microscope was used. Photobleaching was performed by 3 sweeps of a 405 nm laser with 100% power and 100 μs dwell time in the posterior cortex, ingressing furrow and division plane (Figure 3 and Supplementary Figures S5A,B) or 10 sweeps and 40 μs dwell time in the posterior cytoplasm (Supplementary Figure S5C). Acquisition parameters, shutters, focus and photobleaching parameters and regions to be photobleached were controlled using Andor iQ3 software. For central plane imaging in one-cell embryos (Figure 3 and Supplementary Figure S1A), 5 × 1 μm z-stacks were collected in the 488 nm channel every 5 seconds for strains GCP113, GCP21, RZB213 and OD26, and in the 561 nm channel every 5 seconds for strain SWG001. For photobleaching experiments in the posterior cytoplasm (Supplementary Figures S5C,D), 5 × 1 μm z stacks were collected in the 488 nm channel every 0.78 seconds. For cortical dual-color imaging in one-cell embryos, 7 × 0.5 μm z-stacks were collected every 5 seconds for strains GCP21, GCP22, GCP592, and GCP618 (Figures 1, 2 and Supplementary Figures S1, S2), strains GCP21 and GCP113 (Figure 3 and Supplementary Figures S3–S5), and strains GCP22 and GCP927 (Figure 4 and Supplementary Figure S6). For cortical imaging in one-cell embryos of strains RZB213 and SWG001 (Supplementary Figure S1B), 7 × 0.5 μm z-stacks were collected in the 488 nm or 561 nm channels every 5 seconds, respectively.

### Fluorescence Intensity Measurements

Before quantification, each time-lapse movie was corrected for fluorescence intensity decay using the simple ratio method in ImageJ’s bleach correction tool. Quantification of fluorescence recovery after photobleaching in Figures 3A’,E was performed using 3 different ROIs to quantify mean fluorescence intensity over time. ROI 1 is the bleached region (5 × 5 pixel for the posterior cortex and posterior cytoplasm; 5 × 15 pixel for the ingressing furrow and 15 × 200 pixel for the whole division plane). ROI 2 is the whole embryo and corrects for overall photobleaching of the sample resulting from acquisition and photobleaching sweeps (oval: 305 × 205 pixels). ROI 3 is the camera background, measured outside fluorescent regions (20 × 20 pixels). After background subtraction and photobleaching correction, the data was normalized using the double normalization method, and curve fitting was performed to extrapolate the half time of recovery. All operations were performed automatically using easyFRAP software (see below).

For quantification of NMY-2::GFP levels at the equator versus poles in Supplementary Figure S4B, images of the central plane of dividing one-cell embryos were used. A line was traced at the poles and at the equator and the mean intensity was determined after subtraction of the camera background.

### Calculation of Myosin Dissociation Rate

Assuming a diffusion-dominated recovery profile, the myosin dissociation rate (*k*_*off*_) in Figure 3A’ was calculated from FRAP data using the following equation (Bernetti et al., 2019):

(1) $$k_{o\,f\,f}=\frac{\text{log}\,\left(2\right)}{\tau_{1/2}},$$

where τ_1__/__2_ is the half time recovery measured from FRAP fitting curves.

### Cortical Flows Quantification

Cortical flow dynamics were analyzed in one-cell embryos of strains GCP21, GCP22, GCP113, GCP562, GCP618, and GCP927 between anaphase onset and back-to-back membrane configuration. In Figures 2B–E, 4D and Supplementary Figures S2B, S3B,C, S6C, magnitude and direction of cortical flows were quantified using an Iterative Particle Image Velocimetry (PIV) plugin for ImageJ, https://sites.google.com/site/qingzongtseng/piv (Tseng et al., 2011). A 192 × 128 pixel (34.1 × 22.8 μm) region of interest was applied to all embryos to standardize the cortex area and exclude cortical curved peripheries. To average the maximum particle displacement with better resolution, two iterations were performed, in which the application of a larger interrogation window of 64 × 64 pixels (first iteration) was followed by a smaller interrogation window of 32 × 32 pixels (second iteration). Consecutive interrogation windows overlapped by 50%. After flow field generation, the 192 × 128 pixel region was divided in two adjacent sub-regions along the anterior-posterior axis of the embryo for quantification: anterior cortex (1st-108th pixel; 0.2–19.2 μm) and posterior cortex (109th-192nd pixel; 19.4–34.1 μm). Each velocity vector was resolved into components along the anterior-posterior axis, Vx, and the dorsal-ventral axis, Vy. The mean Vx and Vy were estimated for each time point and plotted as a function of time to trace the cortical flow profiles in Figures 2B,C and Supplementary Figures S2B, S3B,C, S6C.

To plot longitudinal flow profiles (Vx) along the anterior-posterior axis in Figures 2D,E, 4D, all vectors along the dorsal-ventral axis with the same x-coordinate (corresponding to the longitudinal position) were averaged and plotted as a function of the position along the anterior-posterior axis.

The cosine similarity analysis measures the correlation between every vector pair of the flow fields as a function of the distance between them for the period of persistent unidirectional flow. A cosine similarity of 1 means the vectors are totally correlated. Cosine similarities between two vectors shown in Supplementary Figure S6B was computed using a custom MATLAB script, according to the following formula:

(2) $$c\,o\,s\,i\,n\,e\,s\,i\,m\,i\,l\,a\,r\,i\,t\,y\,\left(\vec{w_{i},}\,\vec{w_{j}}\right)=\frac{\vec{w_{i}}\cdot \vec{w_{j}}}{||\vec{w_{i}}||||\vec{w_{j}||}}$$

### Calculation of Cortical Compression Rates

Longitudinal cortical compression rates in Figures 3B,C were estimated from cortical flow profiles along the anterior-posterior axis by approximating the spatial derivative for each position using a custom MATLAB script. Cortical compression rates were plotted as a function of both position along the anterior-posterior axis and time relative to anaphase onset in 10 seconds intervals, starting at 25 seconds (when a noticeable region of compression could be identified) and stopping at 95 seconds (start of furrow ingression) after anaphase onset.

### Analysis of Bundle Alignment at the Equatorial Cortex

Deviation from vertical alignment over time in Figure 4B was measured using the Directionality plugin for ImageJ. A region of 30 × 70 pixels corresponding to the furrow region was selected for each movie. The average directionality (angle α, in degrees), was calculated for every frame of each movie using the local gradient orientation method. Deviation from vertical alignment (in degrees) was calculated for each angle α by subtracting the value of this angle from 90°. Absolute numbers were plotted.

### Image Analysis, Quantifications, and Statistics

Image processing and image analysis were performed using Fiji(ImageJ; National Institutes of Health) (Schindelin et al., 2019) and MATLAB (MathWorks). Z-stacks acquired on the cell cortex were projected using Fiji’s maximum intensity projection tool. Images within each panel were scaled equally. Flow kymographs shown in Figure 1D and **Supplementary Figures S3A**, **S6A** were created by tracing a 150 × 5 pixel rectangle positioned at the center of the embryo on the cortical plane and mounted using Fiji’s Make Montage tool, as illustrated in Figure 1D’. Graph plotting, linear regressions and statistical analyses were performed with Prism 6.0 (GraphPad Software). Photobleaching experiments were analyzed using easyFRAP, https://easyfrap.vmnet.upatras.gr (Koulouras et al., 2018). Measurements for cytokinetic analysis in Supplementary Figure S1A were performed as previously reported (Chan et al., 2019). Error bars represent the standard-deviation (SD), standard-error of the mean (SEM), or 95% confidence interval (95% CI), and statistical significance tests were performed using one-way ANOVA with Bonferroni correction, as indicated in figure legends. Statistical significance of flow profiles was performed using two-way ANOVA for repeated measures.

## Data Availability Statement

The raw data supporting the conclusions of this article will be made available by the authors, without undue reservation.

## Author Contributions

AXC and JL: conceptualization. JL: methodology and software. JL, F-YC, DSO, JS, and AFS: validation. JL and F-YC: formal analysis. JL, F-YC, DSO, JS, AFS, and AMS: investigation. JL and AXC: writing – original draft. JL, F-YC, DSO, JS, AFS, AMS, RG, and AXC: writing – review and editing. JL, F-YC, DSO, and AXC: visualization. AXC and RG: supervision. AXC: funding acquisition.

## Conflict of Interest

The authors declare that the research was conducted in the absence of any commercial or financial relationships that could be construed as a potential conflict of interest.

## Supplementary Material

SUPPLEMENTARY FIGURE S1 Delayed cytokinesis in embryos expressing LifeAct::mKate2. **(A)** Intervals for ring assembly/furrow initiation and ring constriction rate (mean ± 95% CI). N is the number of embryos analyzed. Statistical significance was determined using one-way ANOVA followed by Bonferroni’s multiple comparison test. ^****^*P* < 0.0001, ns, not significant. **(B)** Cortical images of embryos expressing different fluorescent probes for actin. Scale bar, 10 μm.

SUPPLEMENTARY FIGURE S2 Rotational flows are a consequence of embryo compression. **(A)** Time projections of control embryos expressing LifeAct::GFP imaged under compression (top) or without compression (bottom). Rotational (Y-component) flows are indicated by curved arrows. Scale bar, 10 μm. **(B)** Rotational (Y-component) flow velocity (mean ± SEM) in the anterior (left) or posterior (right) half of embryos imaged with (black) or without (gray) compression. **(C)** Intervals for ring assembly/furrow initiation and ring constriction rate (mean ± 95% CI) measured in embryos imaged with (black) or without (gray) compression. N is the number of embryos analyzed.

SUPPLEMENTARY FIGURE S3 F-actin and myosin cortical flow profiles are similar. **(A)** Kymographs depicting Lifeact::GFP (top) and NMY-2::GFP (bottom) longitudinal cortical flows from anaphase onset until complete ingression. **(B)** Longitudinal (X-component) and rotational (Y-component) flow velocities (mean ± SEM) for LifeAct::GFP and NMY-2::GFP in the anterior or posterior half of embryos, plotted over time starting at anaphase onset. The depicted interval covers both the period of unidirectional and the period of bidirectional flows. **(C)** Longitudinal (X-component) LifeAct::GFP flow velocities (mean ± SEM) over time for control and NMY-2-depleted embryos from anaphase onset until complete ingression. Arrows indicate average time of shallow deformation (darker tone) or furrow initiation (lighter tone). N is the number of embryos analyzed.

SUPPLEMENTARY FIGURE S4 The amount of myosin that accumulates at the cell equator is higher than the amount that leaves the cortical poles. **(A)** Images of the central and cortical planes of a dividing one-cell embryo expressing NMY-2::GFP at different time points in cytokinesis, revealing a decrease of myosin levels at the cortical poles and an increase at the cell equator. Scale bar, 5 μm. **(B)** Quantification of NMY-2::GFP levels (mean ± SD) on the anterior pole, posterior pole, and equator at the time points indicated in **(A)**, determined as indicated in the schematic on the right. N is the number of embryos analyzed.

SUPPLEMENTARY FIGURE S5 Fluorescence recovery after photobleaching of the entire division plane indicates that myosin continues to accumulate in the contractile ring during furrow ingression. **(A)** Schematic showing the photobleached region of the entire division plane at shallow deformation (top left) and selected images from a time-lapse series showing NMY-2::GFP fluorescence over time pre- and post-bleaching. **(B)** Quantification of NMY-2::GFP fluorescence recovery after photobleaching the entire division plane at shallow deformation. Data from individual embryos (light gray) and the mean ± SD (black) are plotted. **(C)** Quantification of NMY-2::GFP fluorescence recovery after photobleaching in the posterior cytoplasm. **(D)** Boxplots showing the dispersion of half-time recovery for the posterior cytoplasm and the posterior cortex. In each box, the central line is the median, the boundaries of the box are the 25th and 75th percentiles; the whiskers extend to include the maximum and minimum values. N is the number of embryos analyzed. Statistical significance of half-time recovery in (D) was determined using unpaired Student’s *t*-test: ^∗^*P* < 0.05.

SUPPLEMENTARY FIGURE S6 Cortical flows are not aggravated in *nmy-2(RNAi)*;*plst-1(prt89)* embryos. **(A)** Kymographs of LifeAct::GFP cortical flow profiles along the longitudinal axis of the embryos. Yellow, red, and blue bars correspond to contractile ring assembly, furrow initiation, and ring constriction intervals, respectively. Kymographs were built as described in Figure 1D’. **(B)** Cosine similarity between two vectors as a function of the distance between them. **(C)** Longitudinal (X-component) average flow velocity (mean ± SEM) over time after anaphase onset for LifeAct::GFP in the anterior or posterior half of the embryos. Arrows indicate the time of shallow deformation. In panels (B), (C), N is the number of embryos analyzed.

SUPPLEMENTARY TABLE S1 List of *C. elegans* strains used in this study.

SUPPLEMENTARY TABLE S2 List of dsRNAs used in this study.

SUPPLEMENTARY MOVIE S1 Plastin and myosin cooperate to align F-actin bundles at the division plane. Time-lapse series of the cell cortex in dividing one-cell embryos of the indicated genotypes expressing LifeAct::GFP and NMY-2::mCherry. Cortical images are maximum intensity projections of seven z-sections 0.5 μm apart acquired every 5 seconds from anaphase onset (0:00). Frame rate is 6 frames per second, timestamp is minutes:seconds. Scale bar, 5 μm.
