# Supplementary figures and images for: Equatorial Non-muscle Myosin II and Plastin Cooperate to Align and Compact F-actin Bundles in the Cytokinetic Ring
Source: Front Cell Dev Biol. 2020 Sep 25;8:573393. doi: 10.3389/fcell.2020.573393 (PMC7546906; doi:10.3389/fcell.2020.573393)

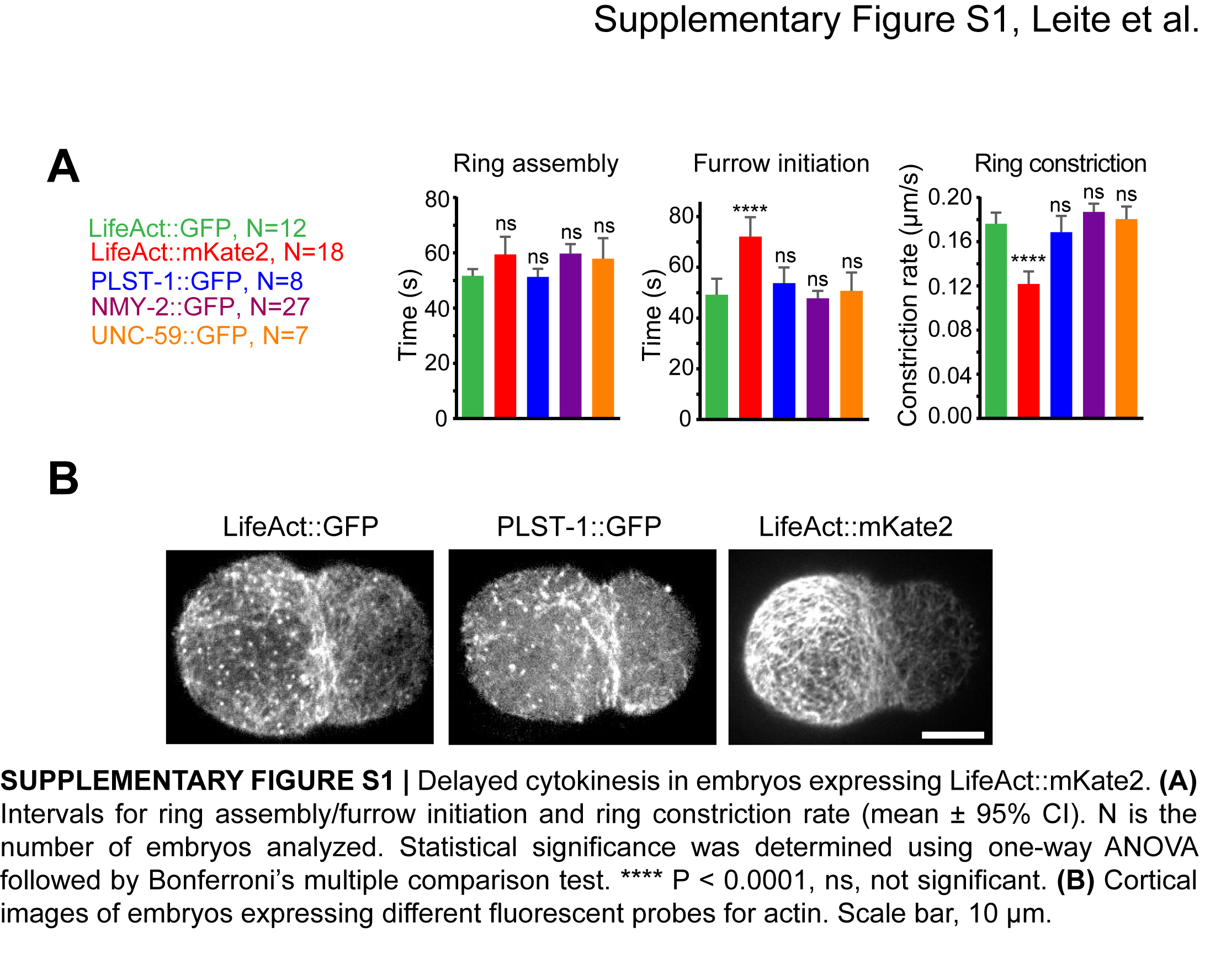

Supplement: SUPPLEMENTARY FIGURE S1 — Delayed cytokinesis in embryos expressing LifeAct::mKate2. (A) Intervals for ring assembly/furrow initiation and ring constriction rate (mean ± 95% CI). N is the number of embryos analyzed. Statistical significance was determined using one-way ANOVA followed by Bonferroni’s multiple comparison test. ****P < 0.0001, ns, not significant. (B) Cortical images of embryos expressing different fluorescent probes for actin. Scale bar, 10 μm. [file Image_1.jpg]

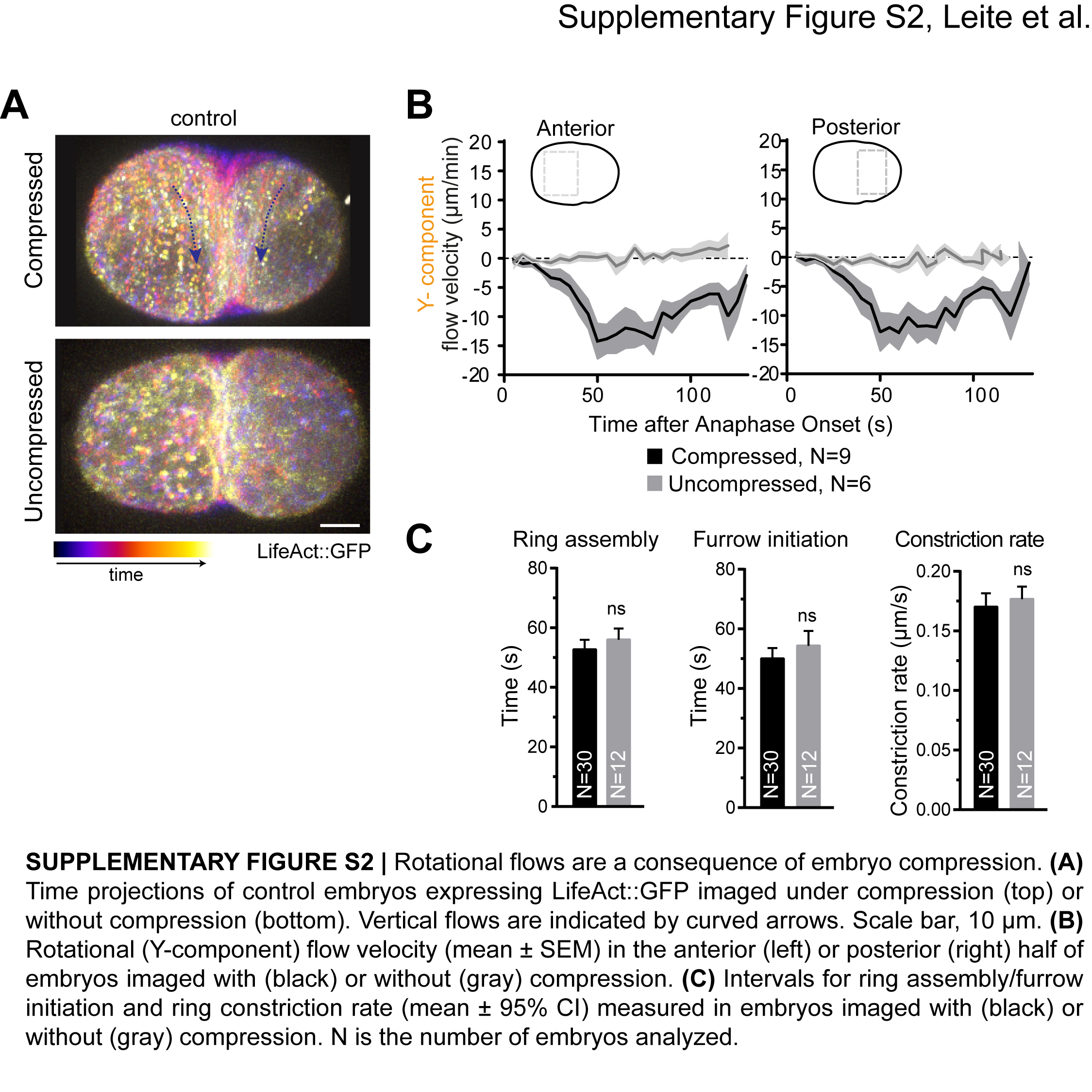

Supplement: SUPPLEMENTARY FIGURE S2 — Rotational flows are a consequence of embryo compression. (A) Time projections of control embryos expressing LifeAct::GFP imaged under compression (top) or without compression (bottom). Rotational (Y-component) flows are indicated by curved arrows. Scale bar, 10 μm. (B) Rotational (Y-component) flow velocity (mean ± SEM) in the anterior (left) or posterior (right) half of embryos imaged with (black) or without (gray) compression. (C) Intervals for ring assembly/furrow initiation and ring constriction rate (mean ± 95% CI) measured in embryos imaged with (black) or without (gray) compression. N is the number of embryos analyzed. [file Image_2.jpg]

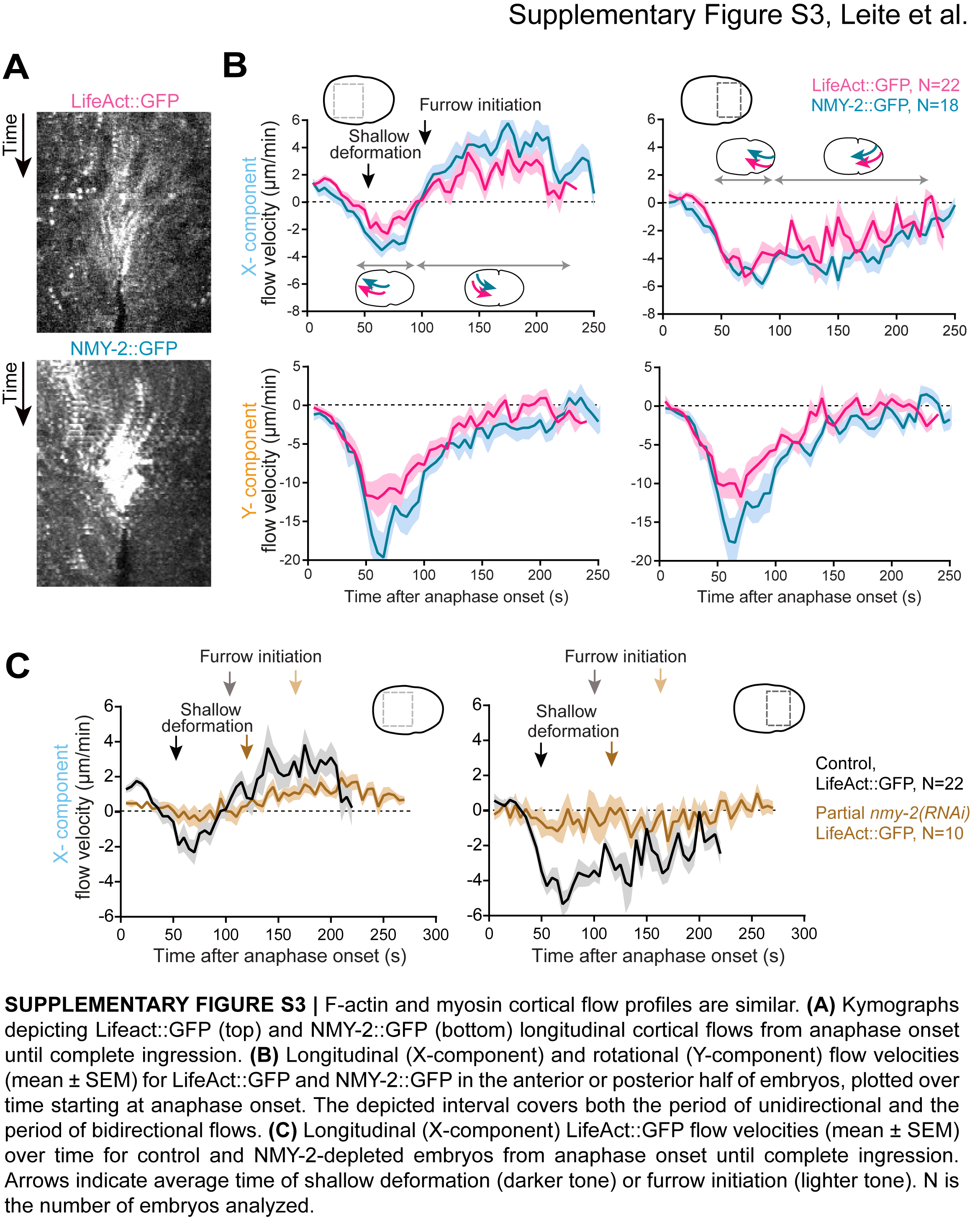

Supplement: SUPPLEMENTARY FIGURE S3 — F-actin and myosin cortical flow profiles are similar. (A) Kymographs depicting Lifeact::GFP (top) and NMY-2::GFP (bottom) longitudinal cortical flows from anaphase onset until complete ingression. (B) Longitudinal (X-component) and rotational (Y-component) flow velocities (mean ± SEM) for LifeAct::GFP and NMY-2::GFP in the anterior or posterior half of embryos, plotted over time starting at anaphase onset. The depicted interval covers both the period of unidirectional and the period of bidirectional flows. (C) Longitudinal (X-component) LifeAct::GFP flow velocities (mean ± SEM) over time for control and NMY-2-depleted embryos from anaphase onset until complete ingression. Arrows indicate average time of shallow deformation (darker tone) or furrow initiation (lighter tone). N is the number of embryos analyzed. [file Image_3.jpg]

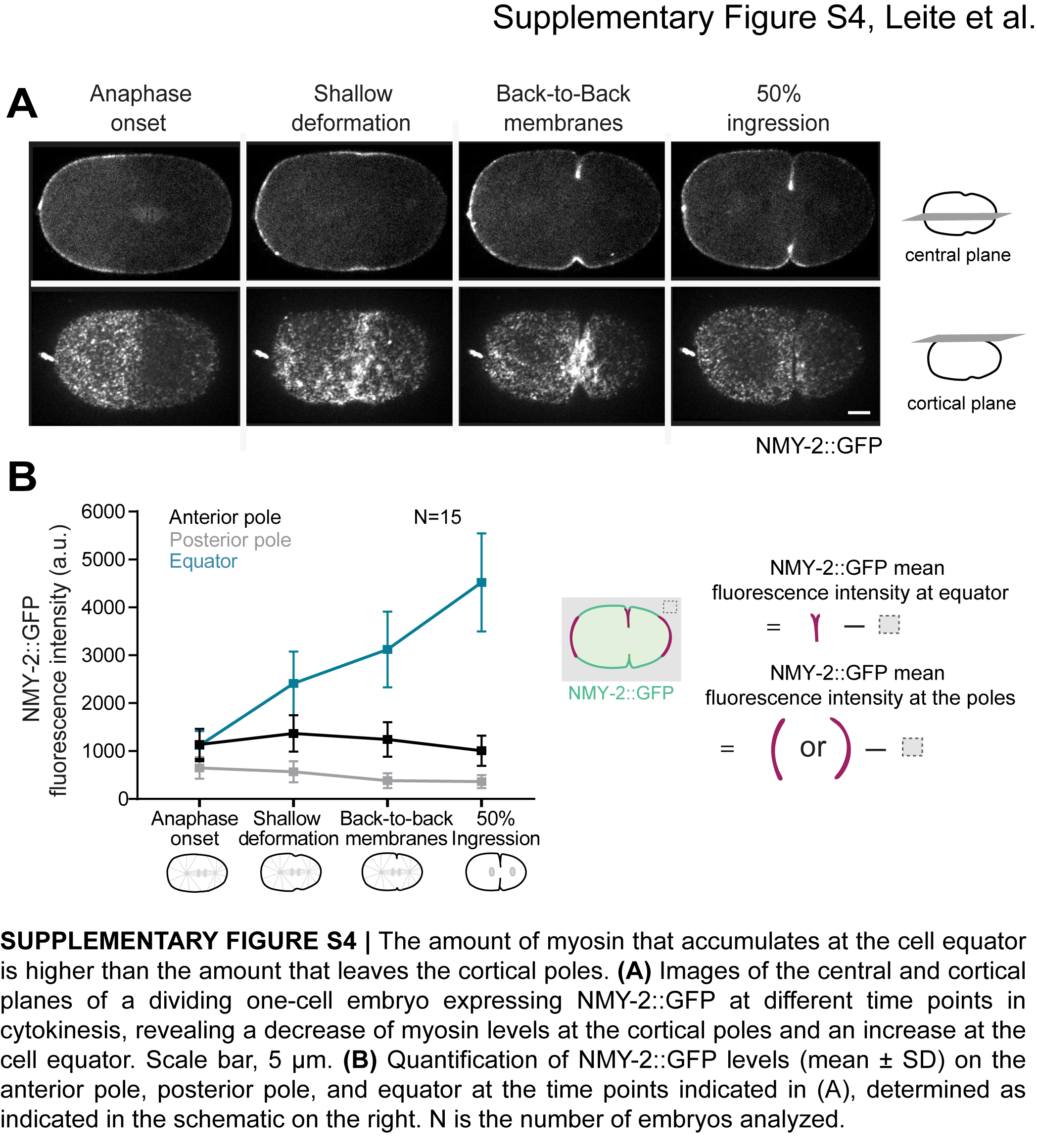

Supplement: SUPPLEMENTARY FIGURE S4 — The amount of myosin that accumulates at the cell equator is higher than the amount that leaves the cortical poles. (A) Images of the central and cortical planes of a dividing one-cell embryo expressing NMY-2::GFP at different time points in cytokinesis, revealing a decrease of myosin levels at the cortical poles and an increase at the cell equator. Scale bar, 5 μm. (B) Quantification of NMY-2::GFP levels (mean ± SD) on the anterior pole, posterior pole, and equator at the time points indicated in (A), determined as indicated in the schematic on the right. N is the number of embryos analyzed. [file Image_4.jpg]

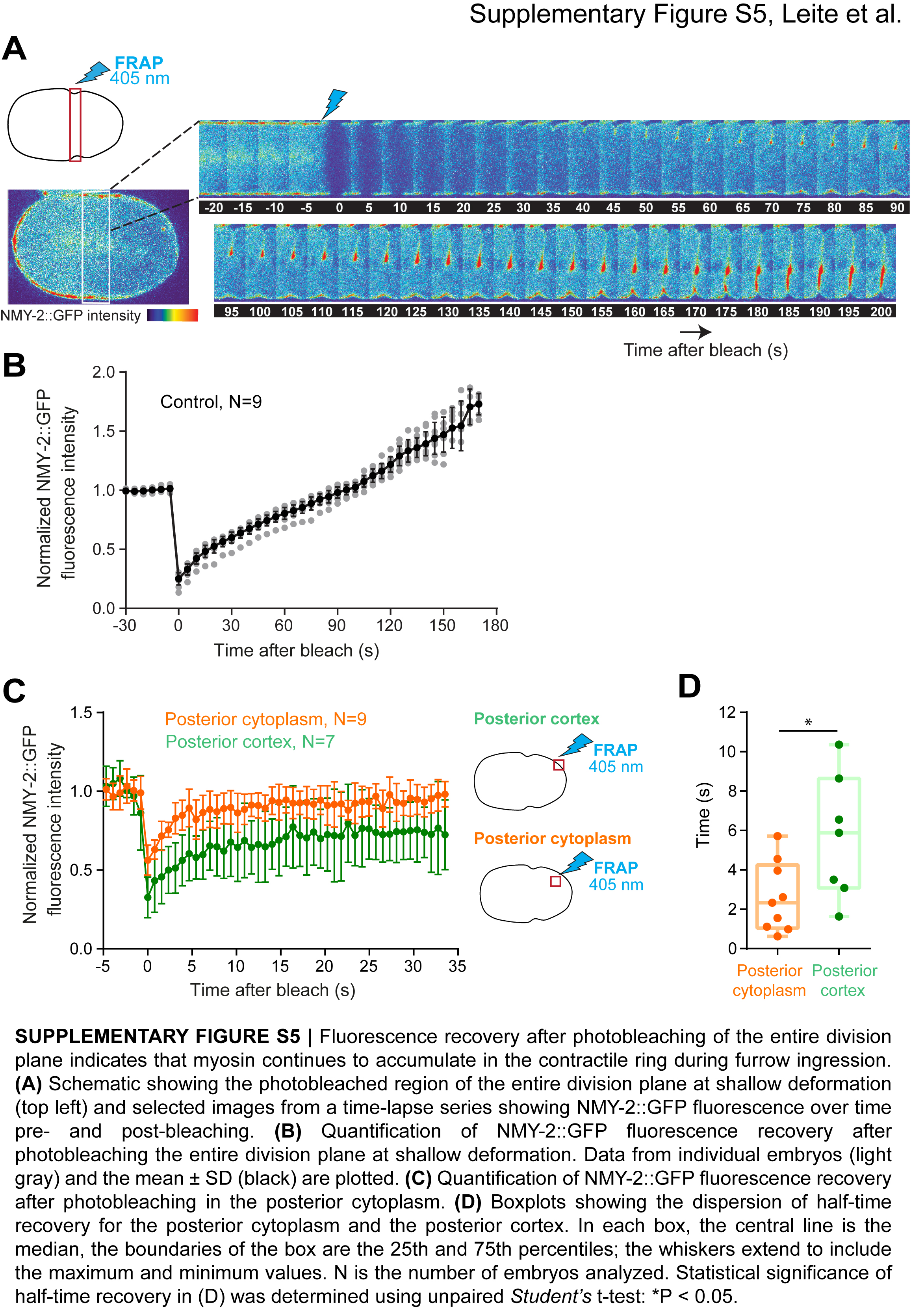

Supplement: SUPPLEMENTARY FIGURE S5 — Fluorescence recovery after photobleaching of the entire division plane indicates that myosin continues to accumulate in the contractile ring during furrow ingression. (A) Schematic showing the photobleached region of the entire division plane at shallow deformation (top left) and selected images from a time-lapse series showing NMY-2::GFP fluorescence over time pre- and post-bleaching. (B) Quantification of NMY-2::GFP fluorescence recovery after photobleaching the entire division plane at shallow deformation. Data from individual embryos (light gray) and the mean ± SD (black) are plotted. (C) Quantification of NMY-2::GFP fluorescence recovery after photobleaching in the posterior cytoplasm. (D) Boxplots showing the dispersion of half-time recovery for the posterior cytoplasm and the posterior cortex. In each box, the central line is the median, the boundaries of the box are the 25th and 75th percentiles; the whiskers extend to include the maximum and minimum values. N is the number of embryos analyzed. Statistical significance of half-time recovery in (D) was determined using unpaired Student’s t-test: ∗P < 0.05. [file Image_5.jpg]

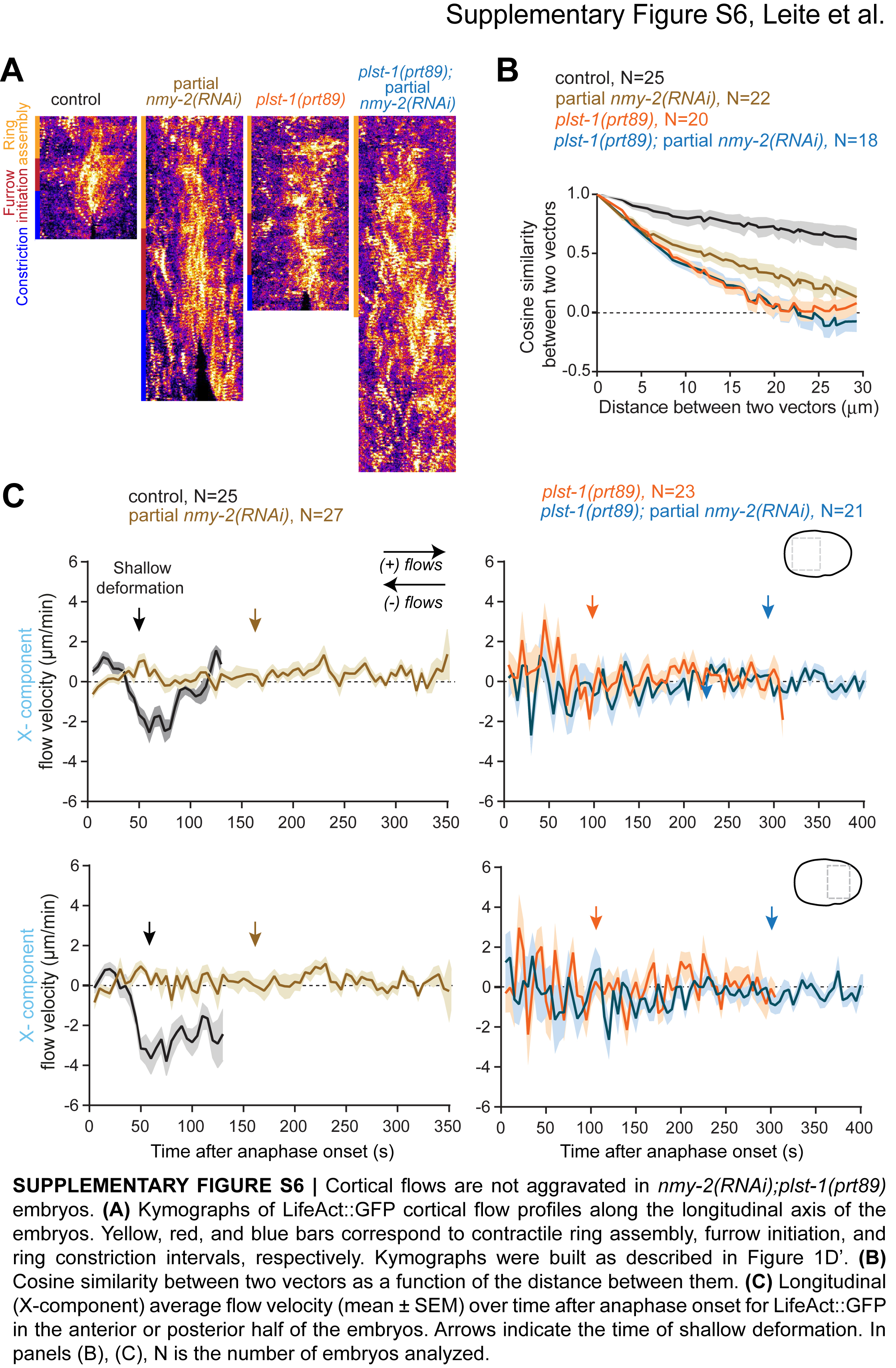

Supplement: SUPPLEMENTARY FIGURE S6 — Cortical flows are not aggravated in nmy-2(RNAi);plst-1(prt89) embryos. (A) Kymographs of LifeAct::GFP cortical flow profiles along the longitudinal axis of the embryos. Yellow, red, and blue bars correspond to contractile ring assembly, furrow initiation, and ring constriction intervals, respectively. Kymographs were built as described in Figure 1D’. (B) Cosine similarity between two vectors as a function of the distance between them. (C) Longitudinal (X-component) average flow velocity (mean ± SEM) over time after anaphase onset for LifeAct::GFP in the anterior or posterior half of the embryos. Arrows indicate the time of shallow deformation. In panels (B), (C), N is the number of embryos analyzed. [file Image_6.jpg]
