# Supplementary material for: Equatorial Non-muscle Myosin II and Plastin Cooperate to Align and Compact F-actin Bundles in the Cytokinetic Ring
Source: Front Cell Dev Biol. 2020 Sep 25;8:573393. doi: 10.3389/fcell.2020.573393 (PMC7546906; doi:10.3389/fcell.2020.573393)
Supplement: SUPPLEMENTARY TABLE S1 — List of C. elegans strains used in this study. [file Table_1.PDF]

Supplemental Table S1 - List of *C. elegans* strains used in this study

Strain	Genotype	Source/ reference
N2	Ancestral	
GCP21	ltIs157 [pAC16;Ppie-1::Life-Act::GFP; unc-119 (+)]; ltIs37 [pAA64; Ppie-1::mCherry::his-58; unc-119 (+)] IV	Our Lab (Silva et al., 2016)
GCP22	ltIs157 [pAC16;Ppie-1::Life-Act::GFP; unc-119 (+)]; unc-119(ed3)III?; prtSi2[pAC71; Pnmy-2::nmy-2reencoded::mCherry::StrepTagII::3'UTRnmy-2; cb-unc-119(+)]II	Our Lab (Osorio et al., 2019)
GCP592	nmy-2 [prt100(R252A)]I; ltIs157 [pAC16;Ppie-1::Life-Act::GFP; unc-119 (+)]; unc-119(ed3)III?; prtSi2[pAC71; Pnmy-2::nmy-2reencoded::mCherry::StrepTagII::3'UTRnmy-2; cb-unc-119(+)]II	Our Lab (Osorio et al., 2019)
GCP618	nmy-2 [prt113(S251A)]I; ltIs157 [pAC16;Ppie-1::Life-Act::GFP; unc-119 (+)]; unc-119(ed3)III?; prtSi2[pAC71; Pnmy-2::nmy-2reencoded::mCherry::StrepTagII::3'UTRnmy-2; cb-unc-119(+)]II	Our Lab (Osorio et al., 2019)
GCP812	nmy-2(cp13[nmy-2::gfp + LoxP]) I; unc-119(ed3) III?; ltIs37 [pAA64; Ppie-1::mCherry::his-58; unc-119 (+)] IV	This study
GCP927	plst-1 [(prt89)]IV; ltIs157 [pAC16;pie-1::Life-Act::GFP; unc-119 (+)]; prtSi2[pAC71; Pnmy-2:nmy-2reencoded::mCherry::StrepTagII::3'UTRnmy-2; cb-unc-119(+)]II; unc-119(ed3)III?	This study
OD26	unc-119(ed3) III; ltIs20 [pASM10; pie-1/GFP::unc-59; unc-119 (+)]	OD Lab
RZB213	[plst-1(msn190[plst-1::GFP])] IV	Zaidel-Bar Lab (Ding et al., 2017)
SWG001	[(Mex5p/lifeact/mKate); unc-119 (+)); unc-119(ed3)] III	Grill Lab (Reymann et al., 2016)
