# Supplementary material for: Equatorial Non-muscle Myosin II and Plastin Cooperate to Align and Compact F-actin Bundles in the Cytokinetic Ring
Source: Front Cell Dev Biol. 2020 Sep 25;8:573393. doi: 10.3389/fcell.2020.573393 (PMC7546906; doi:10.3389/fcell.2020.573393)
Supplement: SUPPLEMENTARY TABLE S2 — List of dsRNAs used in this study. [file Table_2.PDF]

**Table S2 - List of dsRNAs used in this study**

| Name | Gene target | Foward primer | Reverse primer | RNAi |
|---|---|---|---|---|
| nmy-2_RNA#1 | F20G4.3 (nmy-2) | GGCCCGATATCATGAACAACGAGCTTGAAAG | GGCACGATATCAGCCTCCTGGATAGCC | feeding |
| nmy-2_RNA#2 | F20G4.3 (nmy-2) | CCCAAGATATCAATTGAATCTCGGTTGAAGGAA | CCCCCGATATCGACTGCATTTCACGCATCTTATG | feeding |
| nmy-2_RNA#3 | nmy-2:mCherry reencoded (nmy-2::mCherry$^{sen}$) | GGCCCGATATCATGAATAATGAACTCGAGTCAATC | GGCCCGATATCACGTTCTTGAATGGCC | feeding |
